# Causes and consequences of individual variation in animal movement

**DOI:** 10.1186/s40462-020-0197-x

**Published:** 2020-02-17

**Authors:** Allison K. Shaw

**Affiliations:** Department of Ecology, Evolution, and Behavior, University of Minnesota, St. Paul, MN 55108 USA

**Keywords:** Context-dependent, Dispersal kernel, Environmental change, Foraging ecology, Movement ecology, Nomadism, Partial migration, Personality, Plasticity, Population dynamics, Range expansion, Sex-biased dispersal

## Abstract

Animal movement comes in a variety of ‘types’ including small foraging movements, larger one-way dispersive movements, seasonally-predictable round-trip migratory movements, and erratic nomadic movements. Although most individuals move at some point throughout their lives, movement patterns can vary widely across individuals within the same species: differing within an individual over time (intra-individual), among individuals in the same population (inter-individual), or among populations (inter-population). Yet, studies of movement (theoretical and empirical alike) more often focus on understanding ‘typical’ movement patterns than understanding variation in movement. Here, I synthesize current knowledge of movement variation (drawing parallels across species and movement types), describing the causes (what factors contribute to individual variation), patterns (what movement variation looks like), consequences (why variation matters), maintenance (why variation persists), implications (for management and conservation), and finally gaps (what pieces we are currently missing). By synthesizing across scales of variation, I span across work on plasticity, personality, and geographic variation. Individual movement can be driven by factors that act at the individual, population, community and ecosystem level and have ramifications at each of these levels. Generally the consequences of movement are less well understood than the causes, in part because the effects of movement variation are often nested, with variation manifesting at the population level, which in turn affects communities and ecosystems. Understanding both cause and consequence is particularly important for predicting when variation begets variation in a positive feedback loop, versus when a negative feedback causes variation to be dampened successively. Finally, maintaining standing variation in movement may be important for facilitating species’ ability to respond to future environmental change.

## Introduction

Movement is ubiquitous; few organisms die in the exact location they were born having never left. The effects of movement have impacts at almost all ecological levels, affecting moving individuals themselves, shaping the distribution and structure of populations, interspecific and intraspecific interactions, and the flow of nutrients, propagules and pathogens across ecosystems. Animal movement comes in a variety of ‘types’ from daily foraging movements, one-way dispersive movements tied to reproduction, seasonally-predictable round-trip migratory movements, and less predictable erratic nomadic movements [1–3].

One common thread among these movement types is that individuals vary in their movement behavior. Although movement variation is clearly important, it is often viewed as secondary to understanding ‘typical’ movement patterns (Additional file 1), and despite having several frameworks for thinking about movement patterns [2, 3], we lack frameworks for systematically understanding movement variation. Furthermore, studies of movement variation tend to be siloed by the scale where variation occurs: intra-individual studies discuss flexibility (e.g., plastic or conditional strategies), inter-individual studies focus on stable differences (e.g., sex or personality), and inter-population studies refer to population-based differences driven by geographic variation.

The practice of studying the average before variance is representative of other biological topics as well. For plants, predictive models are typically based only on average seed dispersal parameters, and a recent review calls for greater attention to how variance in dispersal influences population and community dynamics [4]. Individual differences have also broadly been ignored in studies of ecological niches until relatively recently [5]. Similarly, most experimental studies of climate change focus on mean change and fail to include extreme climate events (and often inadvertently reduce climate variability as a whole) despite evidence that the future will bring increased climate variability [6].

Other areas of ecology have made strides in incorporating averages and variation. In disease ecology most population-level analyses focus on an average value of infectiousness, yet it is clear that individual differences can play a critical role in disease spread [7]. For example, sexually-transmitted and vector-borne pathogens follow a 20/80 rule with 20% of hosts causing 80% of the transmission events [8]. Thus, there is now recognition of this heterogeneity in the field: the concept of superspreaders, individuals that cause disproportionate amount of new infections [7]. In behavioral ecology, it is recognized that there is behavioral variation both within individuals over time and across individuals. Dingemanse et al. [9] refer to these as ‘plasticity’ and ‘personality’ respectively, arguing that these are complementary aspects of individual behavior and shows how behavioral reaction norms can be used to measure differences in each component as well as their interaction.

**Fig. 1:**
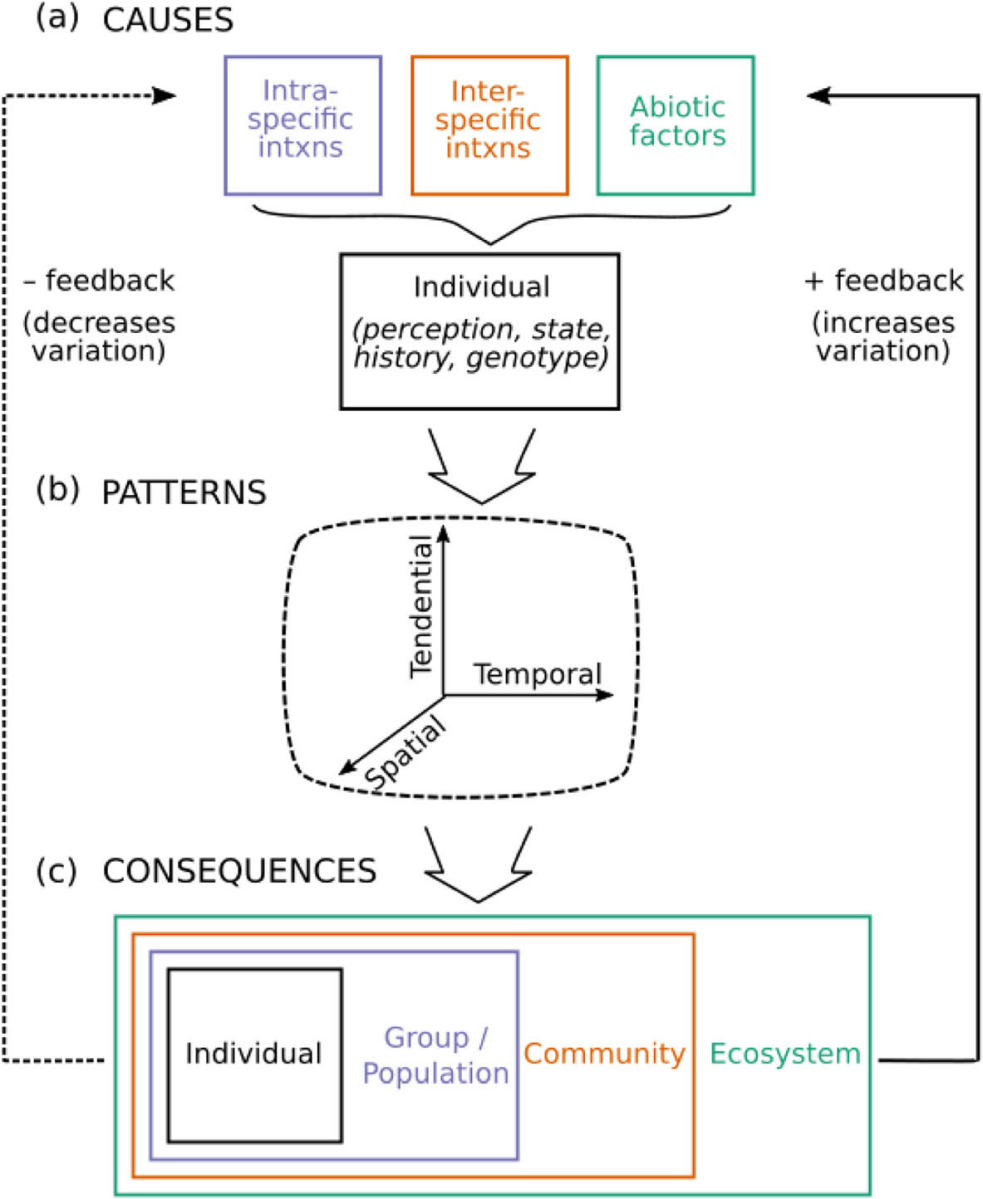
Schematic of the causes, patterns and consequences of movement variation. **a** External factors (environment) are perceived by an individual, and taken in combination with its genotype, internal state and history to determine the movement response, (**b**) movement can vary along three ‘axes’ (whether to move, when to move, and where to move), and (**c**) movement first impacts the individual before potentially scaling up to affect the population, community and ecosystem. While causes often act in parallel, consequences are typically nested. Variation in any of the causes (or their interaction) can contribute to variation in movement, and moving in turn can feed back to affect variation if a consequence of moving is increasing variation in the causes of movement (positive feedback, solid arrow) or decreasing said variation (negative feedback, dashed arrow)

**Fig. 2:**
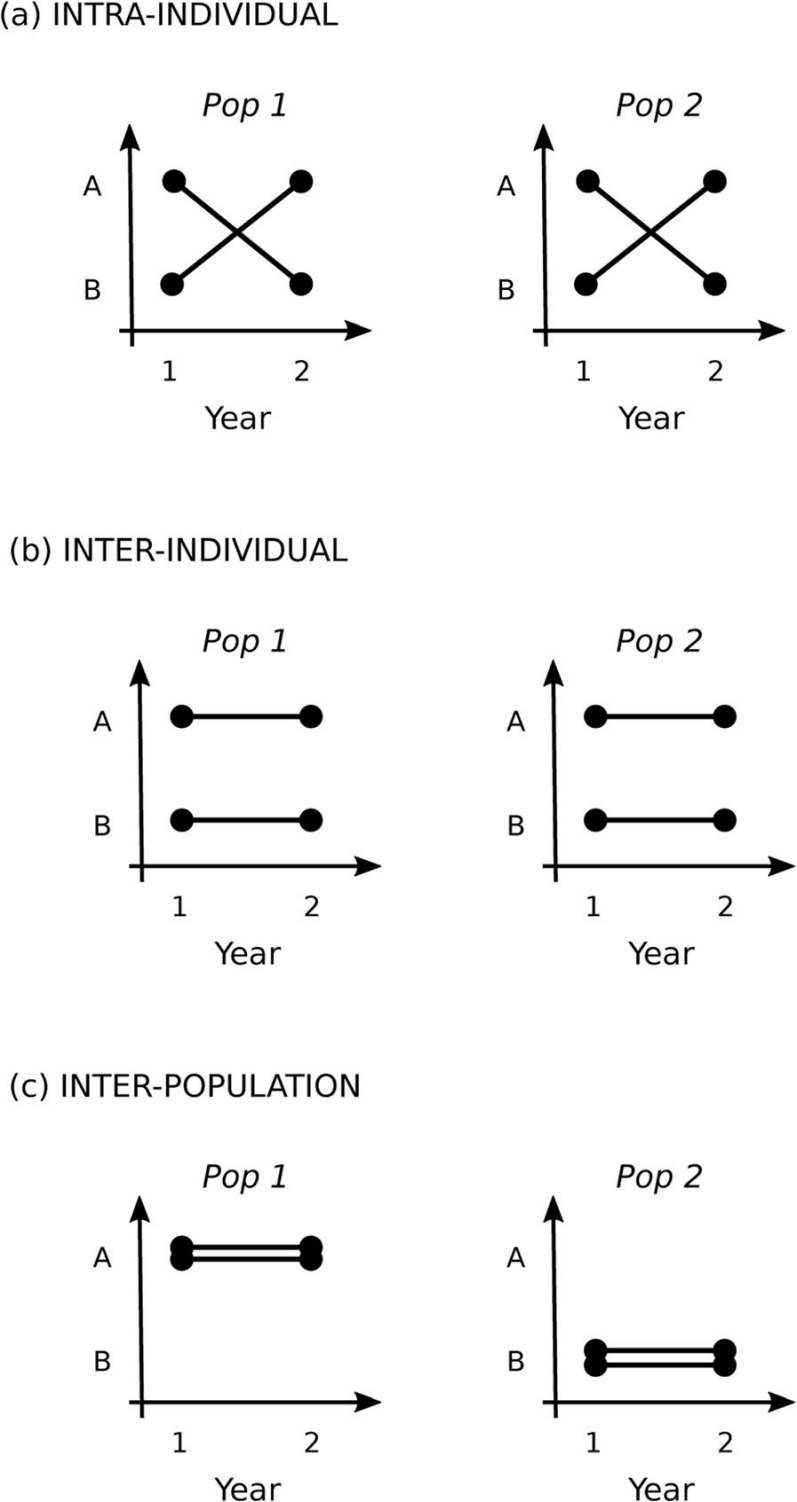
Individual differences in movement can occur at several scales. Each line represent one individual that displays one of two different movement behaviors (A or B) across each of two years, for two different populations. Differences can occur (a) within the same individual over time, (b) among individuals within a population, or (c) among populations of individuals

Here I present an overview of variation in animal movement (Fig. 1, Table 1). I focus on drawing parallels across what I call ‘types’ of movement: dispersal, foraging, migration, and nomadism. Note that these four types are all well-studied (Additional file 1) but are neither exhaustive of all movement (e.g., escape movements) nor mutually exclusive (e.g., an individual may both disperse and migrate at different points in its life). I aim to understand ‘variation’, i.e., differences across individuals within species. Variation can occur at many so-called ‘scales’ (Fig. 2). For example, a single individual may exhibit different movement over its lifetime (intra-individual variation), or individuals with different fixed movement strategies may coexist in the same population (inter-individual), or all individuals within one population may exhibit the same movement strategy, with variation across populations for the species (inter-population). This review encompasses all of these scales. Although many of the patterns described may also apply to interspecific movement variation, that aspect is not covered by this review. Below, I first describe movement variation causes (what factors contribute to individual variation), patterns (what movement variation looks like), and consequences (why variation matters), using non-exhaustive examples from different movement types (dispersal, foraging, migration, nomadism) and scales (intra-individual, inter-individual, inter-population). Finally, I describe movement variation maintenance (how variation persists), implications (for conservation and management), and gaps (what pieces we are currently missing).

**Table 1:** Examples of species with different aspects of movement variation, their causes and consequences

Movement types & System	Variation cause	Variation consequence
Dispersal tendency. Western bluebird (*Sialia mexicana*) populations are currently re-expanding across the United States, coming into contact with a closely related species, mountain bluebirds [10].	Male western bluebirds differ consistently in their level of aggression, with dispersing males more aggressive than philopatric males. More aggressive males are also more likely to win in competitive interactions for territories (even in interactions with mountain bluebirds) but provide little parental care to their offspring (causing high offspring mortality).	Dispersing western bluebirds (who tend to be aggressive) are colonizing into mountain bluebird habitat, outcompeting them, and thus expanding the western bluebird range. Aggressive males are replaced over time by non-aggressive males who produce more surviving offspring and are thus at a selective advantage.
Dispersal distance. Cane toads (*Rhinella marina*) were introduced to Australia in 1935 and have been rapidly spreading across the country [11, 12].	Individuals on the edge of the population range (those that arrive earlier to a new area) move further each day and spend more time dispersing than toads in the center of the population range (those that arrive later).	The spatial assortment by dispersal ability, coupled with a genetic basis for dispersal in cane toads, has caused the population spread to accelerate over time.
Foraging route. Bumble-bees (*Bombus impatiens*) exhibit trapline foraging, repeatedly visiting the same series of plants. Trapline routes differ across individuals and some individuals forage locally instead of traplining [13].	Each bee learns a specific trapline in a few hours, suggesting that different early experiences leads to individual differences in trapline routes.	Bee foraging behavior influences pollination patterns; traplining increases pollen dispersal distance compared to local foraging.
Foraging habitat selection. Spadefoot toad tadpoles (*Spea multiplicatus*)	Tadpoles prefer food associated with their natal habitat/diet.	Related tadpoles with the same natal habitat prefer the same food, leading to associate with kin even in the absence of explicit recognition.
Migration frequency. Pacific leatherback turtles (*Dermochelys coriacea*) spend most of their time in open ocean foraging areas; females migrate every 2–7 years to nesting beaches where they reproduce [14].	Remigration interval (the number of years between successive migrations) varies with local foraging conditions. La Niña years (lower sea surface temperatures) increase the upwelling of nutrient-rich water, leading to faster acquisition of resources for breeding and thus shorter remigration intervals.	Variable remigration intervals leads to variation in annual egg production at the population level.
Migration tendency. Roach (*Rutilus rutilus*) migrate between lakes and streams, driven by seasonal changes in trade-offs between predation risk and forage opportunities (lakes have more food but also more predators) [15, 16].	Individuals with higher body condition are more likely to migrate into streams, foregoing further foraging opportunities in exchange for lower predation risk, while starved fish are more likely to remain in lakes, prioritizing access to food.	Changes in the number of fish migrating from year to year change the predation pressure on zooplankton in lakes, altering zooplankton size structure. Fewer migrating fish lead to a later peak in zooplankton biomass, thus shifting seasonal dynamics of both zooplankton and phytoplankton.

## Causes: what factors contribute to individual differences in movement?

### Intraspecific interactions

Differences in intraspecific interactions can drive individual differences in movement (Fig. 1a). For example, competition can favor increased dispersal at high densities [17, 18]. More nuanced density-dependent dispersal patterns may arise, e.g. when dispersal is tied to locating mates, with males being more likely to settle in areas with low male and high female density [19, 20]. Thus, variation in dispersal may be due in part to variation in local population dynamics. Social hierarchies can lead to differential dispersal or migration based on dominance [21] and social context can drive different migration patterns when migration is socially learned [22]. Social context can also shape foraging strategies in groups where producers (searching for resources) and scroungers (exploiting resources that others find) each do best when they are in the minority, favoring strategy variation at the group level [23]. Finally, differences in position within a population or group can also lead to varaition in movement. For example, group position can be tied to foraging strategy: producers are often at the edge and scroungers in the group core [24, 25]. Similarly, individuals on the edge of a spreading population often are more dispersive than those at the population core [11, 26].

### Interspecific interactions

Differences in interspecific interactions can contribute to individual movement variation, most often at the inter-population scale. Dispersal [27], migration [28] and nomadism [29], can each help individuals escape infection from local build-up of parasites or pathogens. Conversely, infected individuals can be manipulated by their parasites to disperse more [30] or have a higher activity level [31], to increase contact with new hosts. In these cases, individual movement varies based on the local presence/absence of parasites and pathogens. Differences in predation risk can similarly drive differences in dispersal [32] or migration [33], leading to individual movement variation at the species level. On the other side of this relationship, predators can adjust their movement patterns based on their particular prey. For example, individuals that specialize on migratory prey may migrate themselves while individuals that specialize on non-migratory prey are non-migratory [34], causing inter-population movement variation. Finally, differences in food availability can drive whether [35] and when [14, 36] species migrate, and where nomads move [37]. Individuals may also exhibit near-far searching when foraging; moving locally in areas with good forage and moving longer distances otherwise [38].

### Abiotic factors

Differences in local abiotic conditions contribute to individual movement variation (again, often at the inter-population scale; Fig. 1a). Increasingly worse conditions can favor increased movement when moving facilitates escape away from these areas. For example, movement by irruptive nomads can be triggered by an abrupt change in conditions [39], while migration can be driven by seasonally unfavorable conditions like deep snow [40], or rough storms [41, 42]. Variation in these conditions spatially or temporally can lead to variation in movement. Alternatively, increasingly good conditions can favor increased movement when moving facilitates individuals’ ability to make the journey to a new location successfully. For example, moving can be less costly under higher precipitation [43], warmer temperatures [44], or favorable wind directions [45]. More broadly, variation in habitat features [46] can lead to differences in individual movement decisions, while unpredictability in conditions can favor nomadic movements [2].

### Individual traits

Each of the above intra- and inter-specific interactions and abiotic factors are filtered through the individual to shape its movement pattern (Fig. 1a). Variation in any of the causes (or their interaction) can contribute to variation in movement. Movement is thus determined by an individual’s perception of external factors, in combination with its genotype, and internal state (status, history). Some individual traits are more stable while others are more labile.

Traits that are relatively stable contribute to inter-individual differences in movement. For example, sex-specific costs of moving or energetic requirements (associated with reproduction) can lead to sex-biased movement in each dispersal [47], foraging [48], migration [49], and nomadism [50]. The location where an animal was born (natal habitat) can also shape movement decisions through a preference for similar habitat when foraging [51] or dispersing (natal habitat preference induction [52]), or a tendency to return back to the same exact location after migration [53]. Similarly, early life conditions shape which cues an individual uses to navigate [54]. Movement differences can also derive from differences in individual behavioral types where baseline activity, aggression, boldness and sociality affect an individual’s movement ability [55], social foraging tactic [56], tendency to disperse [57] or to migrate [58]. Relatively stable individual differences can either derive from differences in development (i.e., morphs or castes where only some individuals have wings [59], or are gregarious and highly mobile [60]), or be rooted in genetic differences in a movement-related trait [61].

Traits that are more labile (varying through an individual’s lifetime) also contribute to intra-individual variation in movement. For example, costs and benefits to moving can vary with age or life history stage, leading to age- or stage-specific patterns of dispersal [62], nomadism [63], or migration [53]. Individual body size, condition, or degree of hunger/satiation can also contribute to foraging movements [64] as well as tendency to migrate [15, 65, 66] or disperse [67]. Infection status can influence movement at several levels [68] with infected individuals being generally less active [69], less likely to disperse [70], or less likely to migrate [71], or the opposite, with infected individuals actually dispersing more [72]. Finally, movement can vary based on information usage, where individuals migrate following whichever route they first learned [73], or do trapline foraging based on learned habitat patches [13].

## Pattern: what is individual variation in movement behavior?

Individual variation in movement can manifest in different ways (Fig. 1b, Table 1). Variation that is common, often gets referred to with specific terminology (e.g., partial migration). Although terminology can be useful for linking studies within a movement type, this terminology can inhibit cross-talk among studies on different types of movement. Individual variation in movement can be characterized by three ‘axes’: tendential (whether), temporal (when), and spatial (where), common to all types of movement (dispersal, foraging, migration, nomadism).

### Tendential

First, individuals may vary in movement tendency, either with only some individuals moving while others do not, or with variation in how responsive individuals are to stimuli for movement. For example, ‘partial migration’ describes some individuals migrating in a given season while others do not [74, 75], and partial nomadism describes some individuals being nomadic while others are not [39]. In contrast, foraging movements are often named bimodally with individuals either adopting a sedentary (‘sit-and-wait’) or an active (‘search’) strategy [76]. Differences in individual tendency to disperse are described in terms of a dispersal probability or a dispersal rate.

### Temporal

Second, individuals can vary in temporal aspects of movement: the frequency and timing of their movements, and how much time they spend on movement compared to other activities. In some cases, partial migration is driven by differences in individual migration frequency (or staggered frequency), rather than tendency [66]. For group foragers, again there is often a bimodal strategy in movement timing with ‘producers’ actively searching for resources while ‘scroungers’ exploit resources once they are uncovered by others [23].

### Spatial

Finally, individuals can vary in spatial aspects of movement, including distance, direction, area covered, and path tortuosity. These aspects can be quantified by metrics that measure turn angle correlations, stability of home range, and net squared displacement (the distance between an individual’s starting and current locations), as well as final location, which can be quantified in terms of habitat or resource preference [77, 78]. Some species exhibit ‘differential migration’, with individual variation in the distances traveled, often based on individual traits [79, 80]. In other species, individuals starting from the same location differ in their foraging routes with consistent differences in departure angles and terminal points [81]. Individuals can also differ in the distance traveled while foraging or searching; ‘Lévy flights’ describe searching behavior where the distance traveled at each time step varies according to a power law [82]. Variation in dispersal distance is often summarized by a ‘dispersal kernel’, *k*(*x*,*y*), which describes the probability that an individual starting at location *x* will travel to location *y*. Dispersal kernels aggregate variation, either by tracking dispersal at the population level (summing across all individuals and their differences) or by effectively assuming that all individuals are identical but vary in the same way.

## Consequences: why does movement variation matter?

### Individual

At the individual level, variation in movement leads to variation in the costs and benefits in terms of growth, survival and reproduction to individuals (Fig. 1c). All behaviors have costs and benefits; movement is no different. On the whole, if movement is favored by selection, we should expect the benefits of observed movement strategies to outweigh the costs, with the favoring of movement strategies that reduce costs [83]. When individuals differ in their movement, one possible outcome is no net difference in costs and benefits across individuals; e.g., if some individuals experience simultaneously increased costs and increased benefits compared to others. For example, foraging mode influences energy expenditure, where widely foraging individuals both expend and take in more energy than sit-and-wait ones [84]. Alternatively, individuals may trade off costs and benefits across different life history currencies. For example, some migrants trade off the benefit of lower predation risk at the cost of lower foraging opportunities [85] compared to those that stay. Similarly, in species with breeding migrations, migrants trade off survival cost with the benefit of reproduction [66, 86]. A third possibility is that individuals vary in their needs and thus experience costs and benefits differently. For example, pregnant or lactating female mammals have increased energetic needs compared to non-lactating individuals and may adjust their movement patterns accordingly [49, 87].

### Group and population

For group-living species, individual differences can impact group dynamics (Fig. 1c). In highly mobile species, movement variation can challenge group cohesion if individuals travel at different speeds [88] or prefer to move in different directions [89]. Individual heterogeneity in movement can also increase group fragmentation [87] and result in smaller groups, which in turn can affect predation risk and ability to respond to changing environments [90]. Conversely having naive individuals in the same group as individuals with a preferred direction can facilitate decision-making about the direction of travel [89] and facilitate learning of a preferred direction by naive individuals [91]. In more sedentary (colonial) species, variation in movement related to labor partitioning can increase colony efficiency (e.g., eusocial insect castes [92]).

Individual differences in movement also scale up to impact populations. When variation occurs among individuals for a given year (rather than among years for a given individual), movement variation can increase the ‘footprint’ of a population at a snapshot in time. In the extreme, a seemingly stable population distribution can actually be composed of a set of highly nomadic individuals; in any location the identity of individuals changes continuously while the number present remains constant [2]. Given that most dispersing individuals travel short-distances, variation in dispersal distance can be critical for shaping genetic structure and maintaining population connectivity [93, 94]. Thus, movement variation can facilitate gene flow, e.g., if a few individuals occasionally move between otherwise distinct populations to breed [95]. However, if the average dispersal distance is already quite high dispersal variation can decrease inter-patch movements, leading to decreased population connectivity and decreased population size [96]. Individual differences in movement can shape different aspects of population invasion across a landscape, from the probability of colonization (rare long-distance dispersal facilitate introductions [97]), to the dynamics of spread (density-dependent dispersal can cause inter-annual fluctuations in spread [98]), to whether spread occurs at a constant or an increasing rate (fat-tailed kernels and dispersal evolution can accelerate spread [12, 99]). Finally, variation in movement can also lead to variation in infectiousness [100] which can simultaneously lead to higher likelihood of a disease being eradicated as well as larger but rare outbreaks [7].

### Community and ecosystem

Intraspecific movement variation can scale through the population level to impact community and ecosystem processes (Fig. 1c). In contrast, individuals differences in movement that are not detectable at the population level, should be less likely to scale up to impact community or ecosystem dynamics.

Movement variation can have consequences for symmetric pairwise species interactions (e.g. competition, mutualism). For example, variation in dispersal ability linked to behavioral type (e.g., aggression) can increase interspecific competition [10]. However, dispersal variation driven by environmental stochasticity can actually promote the coexistence of two competitors with otherwise identical niches [101]. In terms of mutualisms, pollinator foraging patterns vary with landscape structure, which can in turn impact the pollination rates they provide to plants [102]. Similarly, variation in animal dispersal can drive the spatial patterns of seed rain for animal-dispersed plant species [103].

Movement variation can also have consequences for asymmetric pairwise species interactions (e.g. predator-prey, host-pathogen). For example, many migratory species serve as important prey items for other species [104], so variation in the number of migrants across years could impact food availability and potentially predator population dynamics. Conversely, variation in the number of predators migrating can provide release for prey, leading to changes in both the abundance and seasonal dynamics of prey [16]. Migratory species can also bring parasites and pathogens from diverse locations, leading to outbreaks at stopover sites [105], thus variation in migration timing within species (e.g., degree of synchrony) can drive these infection dynamics [106].

Finally, movement variation can have implications for ecosystem-level processes. Individuals also move around nutrients as they move (foraging in some locations and defecating in others); thus movement variation can cause variable nutrient inputs across ecosystems [107]. As migrants die throughout migration their carcasses become important nutrient sources, and serve as links across ecosystems [108]. Thus variation in migrant numbers across space or over time can lead to nutrient deficits [109].

## Maintenance: what preserves movement variation?

The maintenance of movement variation, like variation in any trait, depends on the level at which variation is encoded. Individual movement differences may be due to different genotypes, in which case they would be maintained by a balance of drift, immigration/emigration, mutation, and selection, as for any other genetic trait. The same genotype may also generate different movement behaviors either across individuals, depending on how they develop (developmental plasticity [110]) or within the same individual over time (activational plasticity [110]). Environmental variation in particular is viewed as major driver of behavioral plasticity, yet behavior itself can ‘construct’ how environmental variation is perceived by an individual [111]. Thus, movement itself feeds back to influence the degree of variation experienced by an individual (Fig. 1, arrows), making disentangling sources of variation a key challenge in understanding movement variation maintenance. Genetic variation in any ‘construction’ trait (such as movement) is also expected to lead to maintenance of plasticity [111].

The fact that movement can simultaneously be affected by and have effects at many different ecological levels (Fig. 1), leaves open the potential for feedback loops between cause and consequence. This is perhaps easiest to see at the individual and population levels. For example, in species that migrate to reproduce, individuals often migrate once they have accumulated enough energetic resources to breed and do not migrate otherwise [66]. Here, migration has a negative feedback (Fig. 1, dashed arrow): by migrating and breeding, individuals expend these resources thus reducing their probability of migrating the next year. As a second example, in a growing and expanding population, individual dispersal variation can lead to spatial sorting, with individuals dispersing the longest distances ending up at the population edge [112, 113]. Assortative mating among these edge individuals may then lead to offspring that disperse even longer distances (The ‘Olympic village effect’; [114]), thus increasing movement variation within the population, in a positive feedback loop (Fig. 1, solid arrow). Examples can also be found at the community and ecosystem level: broadly, environmental predictability shapes movement which in turn can both increase and decrease environmental predictability in feedback loops [115].

Given the role of the environment in shaping movement variation, any change (natural or anthropogenic) to the degree of environment variation stands to influence movement variation. Climate is increasing in variability and current projections show an increase in extreme climate events [116]. Since environmental variation typically begets movement variation, this should increase variability in movement behavior. Anthropogenic disturbances have been shown to increase movement variation by increasing the frequency of switching between traveling and resting [117] and the degree of asymmetry in dispersal (where emmigration and immigration rates differ) [118]. Conversely, other forms of anthropogenic change may decrease environmental variability (e.g., homogenization of conditions through food supplementation), which can shift selective pressures on movement behavior, causing knock-on effects at the population level [119]. Human activities can also move species directly, typically increasing movement variation, by either accidentally introducing alien species [120] or moving individuals as part of an assisted migration conservation strategy [121]. Finally, existing variation in movement behaviors like dispersal can shape the degree to which species are able to respond to environmental change [122].

## Management and conservation implications

What are the conservation and management implications of movement variation? Conservation efforts require knowledge about which habitats should be protected for a given species (e.g., aquatic versus terrestrial [123]), and benefit from knowledge about what relative effort to allocate to different habitat types (within-stream vs ocean-stream connections [124]). Management decisions about networks of protected areas are based on estimates of genetic structure and population connectivity, which are both driven by variation in movement [94, 95, 125]. Understanding the mechanisms linking dispersal variation to population spread can inform eradication efforts for invasive species [126].

The concept of conserving movement strategies has been advocated for in the case of migratory species which provide key ecosystem functions [104] and is likely important for other types of movement. We should consider conserving variation in movement for its own sake. Maintaining standing variation in movement may facilitate species’ ability to track changing climate environments, and improve resilience in responding to anthropogenic change [127]. Since movement variation is an interaction among genotype, individual, and environment (Fig. 1), this may be achievable by conserving both genetic diversity and habitat heterogeneity. It is unclear if there are ways to conserve movement variation driven by individual differences that are independent of genotype and environment; this idea could be explored in future work. A more nuanced conservation strategy would be to first determine at which scale variation is occurring (intra-individual, inter-individual, inter-population) and target strategies accordingly. Minimally movement variation should be accounted for when weighing different strategies. For example, loss of movement variation should be considered one of the impacts of losing genetic diversity.

## Gaps: what are we missing?

I have aimed for this review to be broad – spanning across both types of movement (dispersal, foraging, migration, nomadism) and scale of variation (intra-individual, inter-individual, inter-population). My hope is that researchers interested in variation in only one of these categories will gain new insights by seeing how variation has been considered in other categories.

As a whole, we have a better understanding of the causes of movement variation than of the consequences. Within consequences, the effect of individual movement variation at the community and ecosystem levels is poorly studied compared to its impact at the population level. This may be because individual differences at these higher levels typically need to scale through the population level to have an effect (Fig. 1). That is, individual differences in movement that do not lead to population variation (e.g., only some individuals migrate but the proportion migrating is constant from year to year), are less likely to impact community or ecosystem dynamics. However, this may not always be the case: if individuals differ in certain traits (e.g., stochiometry, consumption rates), movement variation may impact community/ecosystem dynamics even without causing population variation. Thus we have a need for empirical studies to quantify when individual variation affects community and ecosystem processes as well as theoretical studies to predict under what conditions individual variation is most likely to impact community and ecosystem processes. Feedback loops between empirical and theoretical studies will serve to move this research forward as well [4].

As a first step, reporting different forms of variation in collected data (e.g. both the standard deviation in movement traits as well as outliers), will contribute to a broader scientific culture of accounting for movement variation [4]. Second, collected data can be used to account for variation within and across classes of individuals simultaneously, e.g., by characterizing male and female dispersal kernels [128], rather than a single dispersal kernel or sex-specific fixed dispersal distances. Finally, future studies should consider interactions between variation at different scales, starting with experimental design and analysis. For example, the framework of behavioral reaction norms from behavioral ecology generally [9] can be used to look at interactions between intra-individual and inter-individual differences in movement ecology specifically.

Future theoretical studies could help shape the way we think about movement variation in several ways. First, theoretical frameworks could be developed to determine whether there are certain characteristics of a biological system or scenario that could be used to predict when movement variation is most likely to be important. Such frameworks could help guide which future empirical studies should focus on variation and when it can safely be ignored. Second, theory could be developed to predict when variation begets more variation versus when it has a dampening effect (Fig. 1). Although movement variation should be relatively easy to implement in theoretical studies, most models take a simplistic approach to variation [90]. Thus, using models to explore a greater diversity of variation forms could greatly improve our understanding of the causes and consequences of movement variation. A major challenge in future theoretical work is developing techniques that enable models to capture variation without sacrificing too much analytic tractability, which reduces interpretability [129]. For example, current theory can account for stable individual differences or labile individual strategies separately, but accounting for both is most often done via individual-based models which can make nuanced interpretations challenging.

Future empirical studies could help fill gaps in our knowledge of existing movement variation. Across movement types, we have a better understanding of variation in dispersal and migration than we do for nomadism (Table 1); somewhat ironic given the perception of nomadism as a highly variable movement pattern. We also have a better conceptual understanding of variation in negative interspecific causes and consequences (e.g., predator-prey, host-parasite) than in positive interspecific interactions (e.g., mutualisms, facilitation), mirroring a broader trend in interspecific interaction studies [130]. Empirical work that compares movement variation in lab-based and field-based studies can help us understand when there are positive vs negative feedback loops in variation. One of the appeals of lab system is that they present a more controlled (and less variable) environment. Thus, comparing these will determine when effects measured in the lab will be amplified (versus dampened) in a variable field setting, helping to extrapolate from lab-based studies on variation to generate field-based predictions.

## Conclusions

Individual variation in movement is almost as ubiquitous as movement behavior itself. However, our understanding of the causes and consequences of movement variation lags far behind our understanding of ‘typical’ movement patterns, in part because understanding differences requires more data and longer-term studies than understanding averages. Papers in the past few years have called for a better understanding of individual differences impacting movement in terms of personality [55] and collective movements [90]. Here, I have highlighted the need to understand variation in animal movement at all scales (including these) and I have presented a framework (Fig. 1) for thinking about movement variation that draws parallels across different movement types (dispersal, foraging, migration, nomadism). Unfortunately, the areas where variation is understudied are particularly those areas where movement variability can have critical impacts: at community and ecosystem levels. This lack of understanding is likely because the consequences of movement variation are nested across levels, with individual differences only having an indirect impact at the community and ecosystem level, acting via their impact on the population level (Fig. 1). Developing theory that explores a broader range of variation in movement patterns could be especially useful in understanding consequences across these levels. This review, in conjunction with a similar recent call from the plant perspective [4] highlights the immediate need to understand how individual movement differences scale up beyond the population level, across all kinds of organisms.

## Supplementary information

**Additional file 1.** Trends in studying movement variation over time.

## Data Availability

Not applicable.
