# Supplementary material for: Causes and consequences of individual variation in animal movement
Source: Mov Ecol. 2020 Feb 17;8:12. doi: 10.1186/s40462-020-0197-x (PMC7027015; doi:10.1186/s40462-020-0197-x)

**Additional file 1.** Trends in studying movement variation over time.

To get a sense of the relative study of variation in each movement type compared to overall study of that type, I conducted a series of Web of Science searches and counted the number of papers by publication year for each. To get a base-line of the number of papers on each movement type, I searched for each “migration”, “foraging”, “dispersal”, and “nomadism” as topics. Next, I used two approaches to roughly estimate the studies for each type that looked at variation. First, I searched for each movement type plus “variation” (e.g., “foraging variation”) as the topic. Second, I used the most common specialized term that indicates variation for each movement type: “partial migration”, “dispersal kernel”, and “partial nomadism” (I could not find a single common specialized term for variation in foraging).

Next, I looked at the percent of studies within each type that looked at variation using each of the two methods (e.g., for each publication year, I divided the number of studies on “partial migration” by the number of studies on “migration”, and then I divided the number of studies on “migration variation” by the number of studies on “migration”). This revealed two interesting patterns. First, only a small percent of studies looked at variation for any movement type (1-18%, Table A). Second, the study of movement variation has changed over time. The use of “variation” is really only common in the last 30 years, with similar trends for all movement types (Figure A top). The use of special terms has changed both over time and across movement types; “partial migration” was used even in the early 1900s whereas “dispersal kernel” and “partial nomadism” are only found in the last 20 years (Figure A bottom), and all three terms have been relatively common most recently.

**Table A**

| **Movement Type** | **Overall** | **“Variation”** | **Specialized Term** |
| --- | --- | --- | --- |
| migration | 481828 | 22263 [5%] | 7491 [2%] |
| foraging | 100431 | 12629 [13%] | NA |
| dispersal | 84122 | 14747 [18%] | 907 [1%] |
| nomadism | 882 | 43 [5%] | 16 [2%] |

**Figure A**

The percent of studies of each movement type that looked at movement variation over time as quantified by: (top) the number of studies with “X variation” in the topic divided by the number of studies with “X” in the topic, and (bottom) the number of studies with specialized terms (“partial migration”, “dispersal kernel”, “partial nomadism”) in the topic divided by the number of studies with the movement type (“migration”, “dispersal”, “nomadism”) in the topic.


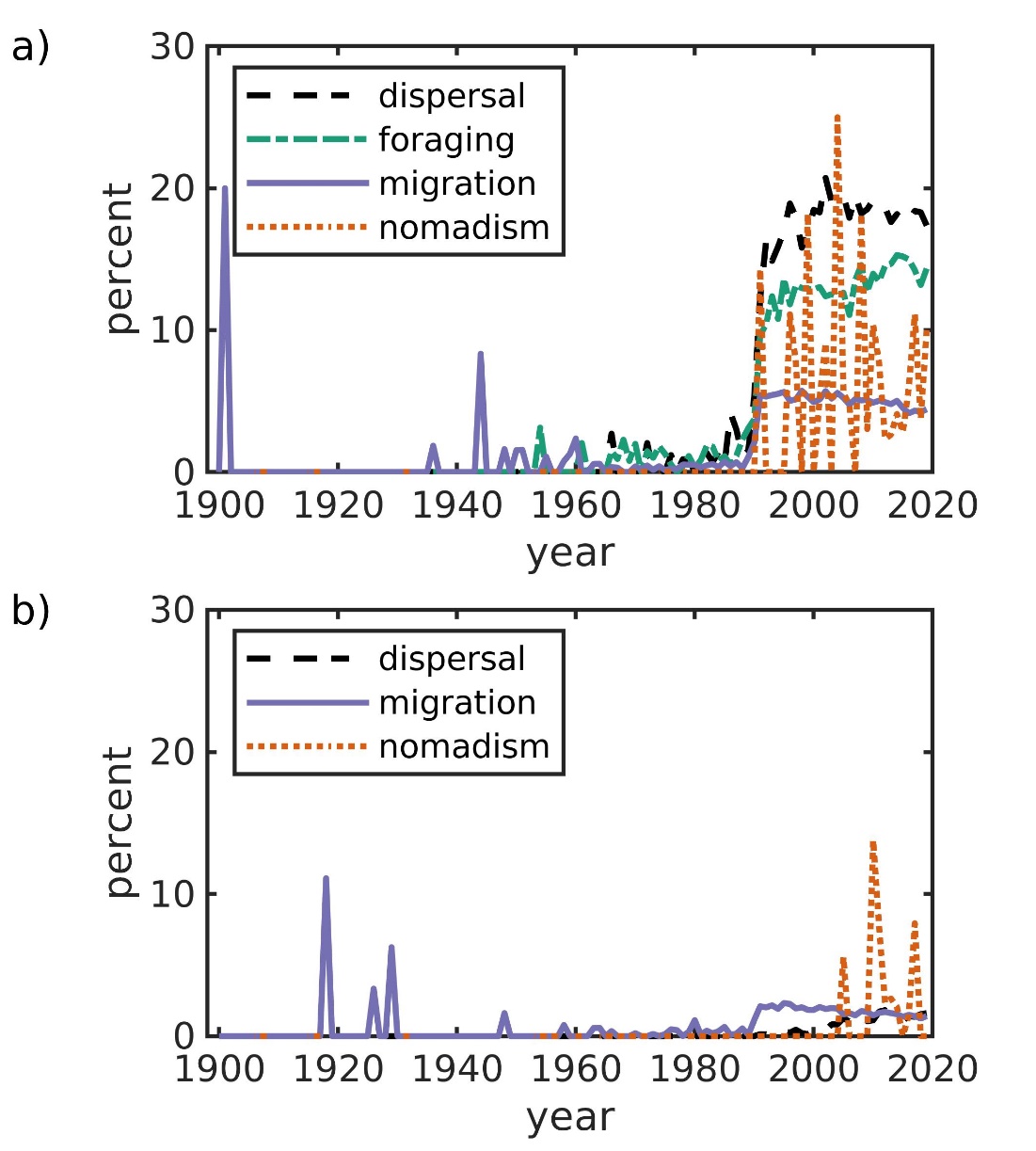

Supplement: Supplementary file 1 — Additional file 1. Trends in studying movement variation over time. [file 40462_2020_197_MOESM1_ESM.docx]
